# Influenza Virus Infection Induces the Nuclear Relocalization of the Hsp90 Co-Chaperone p23 and Inhibits the Glucocorticoid Receptor Response

**DOI:** 10.1371/journal.pone.0023368

**Published:** 2011-08-10

**Authors:** Xingyi Ge, Marie-Anne Rameix-Welti, Elyanne Gault, Geoffrey Chase, Emmanuel dos Santos Afonso, Didier Picard, Martin Schwemmle, Nadia Naffakh

**Affiliations:** 1 Institut Pasteur, Unité de Génétique Moléculaire des Virus à ARN, Département de Virologie, Paris, France; 2 CNRS, URA3015, Paris, France; 3 Université Paris Diderot, Sorbonne Paris Cité, Unité de Génétique Moléculaire des Virus à ARN, Paris, France; 4 Université Versailles Saint-Quentin-en-Yvelines, Guyancourt, France; 5 Department of Virology, Institute for Medical Microbiology and Hygiene, University of Freiburg, Germany; 6 Département de Biologie Cellulaire, Université de Genève, Genève, Switzerland; University of Cambridge, United Kingdom

## Abstract

The genomic RNAs of influenza A viruses are associated with the viral polymerase subunits (PB1, PB2, PA) and nucleoprotein (NP), forming ribonucleoprotein complexes (RNPs). Transcription/replication of the viral genome occurs in the nucleus of infected cells. A role for Hsp90 in nuclear import and assembly of newly synthetized RNA-polymerase subunits has been proposed. Here we report that the p23 cochaperone of Hsp90, which plays a major role in glucocorticoid receptor folding and function, associates with influenza virus polymerase. We show that p23 is not essential for viral multiplication in cultured cells but relocalizes to the nucleus in influenza virus-infected cells, which may alter some functions of p23 and Hsp90. Moreover, we show that influenza virus infection inhibits glucocorticoid receptor-mediated gene transactivation, and that this negative effect can occur through a p23-independent pathway. Viral-induced inhibition of the glucocorticoid receptor response might be of significant importance regarding the physiopathology of influenza infections in vivo.

## Introduction

The genome of influenza A viruses consists of eight molecules of single-stranded RNA of negative polarity. The viral RNAs (vRNAs) are associated with the nucleoprotein (NP) and with the three subunits of the polymerase complex (PB1, PB2 and PA) to form viral ribonucleoproteins (vRNPs) (reviewed in [1]). Once in the infected cells, the vRNPs are transported to the nucleus, where they undergo transcription and replication. Newly synthetised NP and polymerase subunits are imported from the cytoplasm into the nucleus to form new vRNPs. At late stages in infection, vRNPs are exported from the nucleus to the cytoplasm, and assembly with the other viral proteins occurs at the plasma membrane. There are evidence for physical and functional association between the vRNP components and the cellular machineries for transcription, nuclear import and nuclear export. A model for the import of newly synthetised polymerase has been proposed, based on the findings that the RanBP5 importin interacts with the PB1-PA dimer [2], and importins α interacts with PB2 [3], [4]. The Hsp90 protein was found to interact with both PB1 and PB2 and to undergo nuclear relocalization in infected cells [5], suggesting that it could also be involved in nuclear import of newly synthetised viral polymerase subunits. Nuclear proteins are clearly involved in the production of viral RNAs. In particular, the synthesis and processing of viral messenger RNAs (mRNAs) depends on cellular mRNAs transcription [1], [6], [7], [8], [9], [10], splicing [11] and export [12], [13] machineries. Nuclear export of the newly synthetized vRNPs is promoted by the M1 and NEP viral proteins and mediated by molecular interactions with the cellular CRM1 export pathway [14], [15]. Further characterization of the interplay between vRNPs and host factors is needed for a better understanding of the molecular mechanisms of viral RNAs synthesis and trafficking in the host cell, and the role of the RNA polymerase as a determinant of influenza virus host range and pathogenicity. In the longer term, it could provide a rationale for the development of antivirals targeting essential interactions between vRNPs and host factors.

Here we used a recombinant influenza virus expressing a PB2 protein fused to a purification tag to identify vRNP-associated host factors. We report that the p23 cochaperone of Hsp90, which plays a major role in the folding and function of glucocorticoid receptors [16], associates with the viral polymerase and relocalizes to the nucleus in influenza virus-infected cells. We show that p23 is not essential for viral multiplication in cultured cells, and that glucocorticoid receptor-mediated signalling is impaired in influenza virus-infected cells.

## Materials and Methods

### Plasmids

The series of eight pPolI plasmids containing the sequences corresponding to the genomic segments of WSN virus, and the four recombinant pcDNA3.1 plasmids for the expression of WSN-PB1, -PB2, -PA and -NP proteins [17] were kindly provided by G. Brownlee (Sir William Dunn School of Pathology, Oxford, UK). In order to insert the Strep-tag sequence downstream the PB2-ORF into the pPolI-PB2 plasmid, two PCR reactions were performed in parallel, using pPolI-PB2 or pEXPR-IBA103 (IBA GmbH) as a template, and oligonucleotides designed so that the amplified products contained an overlapping sequence corresponding to the junction between the PB2 and Strep-tag coding sequences 5′-TGGATTATCAGAAACTGGGAAAC-3′ and 5′GTCGTCATCGTCTTTGTAGTCAGCTGCATTGATGGCCATCCGAATTCTTTTGGTCG-3′ on the one hand, 5′-CATCAATGCAGCTGACTACAAAGACGATGACGACAAATAGTGTCGAATAGTTTAAAAACGACCTTG-3′ and 5′-CAGCTGGCGAAAGGGGGATGTGC-3′ on the other hand). An equimolar mix of the amplified products was used as a template for a third PCR reaction, and the resulting amplicon was cloned between the NheI and BstXI sites of plasmid pPolI-PB2-Flag-143 [18]. The same protocol was used for insertion of the HA tag sequence downstream the PB2-ORF into the pPolI-PB2 plasmid. The pPR7-FluA-Luc plasmid was constructed by replacing the sequences encoding CAT by the sequences encoding the *Renilla* luciferase in the pPR7-FluA-CAT plasmid [19], using a standard PCR-based protocol.

The GR expression vector and GR luciferase reporter plasmid were described previously [20].

The p23 and EF1α coding sequences were amplified from a human spleen cDNA library (kindly provided by Y. Jacob, Institut Pasteur, Paris) with the oligonucleotides 5′-GGGGACAACTTTGTACAAAAAAGTTGGCATGCAGCCTGCTTCTGCAAAGTGGTACGATC-3′ and 5′-GGGGACAACTTTGTACAAAAAAGTTGGCAGTTACTCCAGATCTGGCATTTTTTCATCATCAC-3′ for p23, and 5′-GGGGACAACTTTGTACAAAAAAGTTGGCGGAAAGGAAAAGACTCATATCAACATTGTCG-3′ and 5′-GGGACAACTTTGTACAAAAAAGTTGTTAGTTATTTAGCCTTCTGAGCTTTCTGGGCAG-3′ for EF1α). The resulting amplicons were subcloned using the Gateway technology downstream the GST coding sequences into a pCMV-GST plasmid (Y. Jacob, Institut Pasteur, Paris), for construction of the pCMV-GST-p23 and pCMV-GST-EF1α plasmids.

All constructs were verified by the sequencing of positive clones using a Big Dye terminator sequencing kit and an automated sequencer (Perkin Elmer). The sequences of the oligonucleotides used for amplification and sequencing can be obtained upon request.

### Cell and viruses

Wild-type and p23^−/−^ MEFs [21], 293T (ATCC, CRL-11268) and A549 (ATCC, CCL-185) cells were grown in complete Dulbecco's modified Eagle's medium (DMEM) supplemented with 10% fetal calf serum (FCS). MDCK cells were grown in modified Eagle's medium supplemented with 5% FCS.

The method used for the production of the WSN-PB2-Strep and P908-WSN-PB2-Strep recombinant influenza viruses by reverse genetics was adapted from previously described procedures [17], [18]. Briefly, the eight pPolI and 4 pcDNA3.1 plasmids (0.5 µg of each) were co-transfected into a subclonfluent monolayer of cocultivated 293T and MDCK cells (4×10^5^ and 3×10^5^ cells, respectively, in a 35-mm dish), using 10 µl of the Fugene 6 transfection reagent (Roche). After 24 hours of incubation at 35°C, the supernatant was removed and replaced with DMEM supplemented with 2% FCS, and the cells were incubated at 35°C for two more days. The efficiency of reverse genetics was evaluated by titrating the supernatant on MDCK cells, in a standard plaque assay using an agarose overlay in complete MEM with 2% FCS. Viral stocks were produced by infecting MDCK cells at a m.o.i. of 0.001 and collecting the supernatant after an incubation of 2 days at 35°C in DMEM supplemented with 2% FCS. Experimental infections were performed at 37°C, unless otherwise indicated.

### PB2-strep complex purification on Strep-tactin colums

At 6 hours following infection of 293T cells (4×10^8^ cells per 150 mm dishes) with the PB2-wt or PB2-Strep viruses at a m.o.i. of 5 pfu/cell, cells were washed twice with PBS, collected with a cell scraper and centrifuged at 450 g for 5 min. The packed cell volume (PCV) was estimated. Cells were resuspended in 5×PCV of a hypotonic lysis buffer (Hepes 100 mM, MgCl_2_ 1.5 mM, KCl 100 mM, DTT 1 mM, Protease Inhibitor Cocktail-Sigma) and kept on ice for 15 mn. NP40 was added at a final concentration of 0.3%, and the lysate was centrifuged at 11,000 g for 2 mn at +4°C. The supernatant corresponding to the cytoplasmic fraction was transferred to a fresh tube, 1∶20 of the volume was frozen at −80°C. The remaining volume was loaded on a 1 ml Strep-tactin column following the recommendations of the supplier (IBA GmbH). Following elution with 6×200 µl of a desthiobiotin solution (IBA GmbH), elution fractions n° 2 to 5 were pooled and concentrated approximately 15-fold using a 10,000 MWCO Vivaspin tube (Sartorius). One third of the resulting sample was subjected to electrophoresis on a 4–15% Tris-Glycine-SDS polyacrylamide gel (Biorad). Following overnight staining of the gel with SYPRO-Ruby (Invitrogen), or western-blotting as described below, the proteins were vizualized using the G-Box (Syngene).

### Mass spectrometry analysis

Destaining of SYPRO-Ruby-stained gel slices, reduction, alkylation, trypsin digestion of the proteins followed by peptide extraction were carried out with the Progest Investigator (Genomic Solutions). Peptides were eluted directly using the ProMS Investigator, (Genomic Solutions) onto a 96-well stainless steel MALDI (Matrix Assisted Laser Desorption Ionisation) target plate (Applied Biosystems) with 0.5 µL of CHCA (alpha-cyano-4-hydroxy cinnamic acid) matrix (2,5 mg/ml in 70% Acetonitrile, 30% H_2_O, 0.1% Trifluoroacetic acid).

Raw data for protein identification were obtained on the 4800 Proteomics Analyzer (Applied Biosystems) and analyzed by GPS Explorer 2.0 software (Applied Biosystems/MDS SCIEX). For positive-ion reflector mode spectra 3000 laser shots were averaged. For MS calibration, autolysis peaks of trypsin ([M+H]^+^ = 842.5100 and 2211.1046) were used as internal calibrates. Monoisotopic peak masses were automatically determined within the mass range 800–4000 Da with a signal to noise ratio minimum set to 20. Up to 10 of the most intense ion signals were selected as precursors for Tandem Mass Spectrometry (MS/MS) acquisition excluding common trypsin autolysis peaks and matrix ion signals. In MS/MS positive ion mode, 4000 spectra were averaged, collision energy was 2 kV, collision gas was air and default calibration was set using the Glu^1^-Fibrino-peptide B ([M+H]^+^ = 1570.6696) spotted onto fourteen positions of the MALDI target. Combined Peptide Mass Fingerprinting (PMF) and MS/MS queries were performed using the MASCOT search engine 2.1 (Matrix Science Ltd.) embedded into GPS-Explorer Software 3.5 (Applied Biosystems/MDS SCIEX,) on the NCBInr [20100119 (10348164 sequences; 3529470745 residues)] database with the following parameter settings: 50 ppm mass accuracy, trypsin cleavage, one missed cleavage allowed, carbamidomethylation set as fixed modification, oxidation of methionines was allowed as variable modification, MS/MS fragment tolerance was set to 0.3 Da. Protein hits with MASCOT Protein score ≥83 and a GPS Explorer Protein confidence index ≥95% were used for further manual validation.

### Affinity purification of GST-fusion proteins

Plasmids pCMV-GST-p23, pCMV-GST-EF1α or pCMV-GST (1 µg) were transfected together with pcDNA3.1-PB1, -PB2, and/or -PA (1 µg) into a subconfluent monolayer of 293T cells (8×10^5^ cells in a 35-mm dish) using 10 µl of the Fugene-6 transfection reagent (Roche). At 48 hours post-transfection, cells were lysed in 300 mL of Tris-HCl [pH 7.4] 50 mM, NaCl 120 mM, EDTA 1 mM, NP40 1% and Protease Inhibitor Cocktail 1X (Sigma). Gluthation Sepharose 4 Fast Flow beads (GE Healthcare) were added to the supernatant and incubated overnight under gentle rotation. Beads were washed three times in Tris-HCl [pH 7.4] 50 mM, NaCl 120 mM, EDTA 1 mM, NP40 1%, and bound proteins were eluted by incubation with 30 µl of Laemmli buffer.

### siRNA transfection,viral-minigenome and GR-mediated gene transactivation assay

Subconfluent monolayers of 293T cells (2×10^5^ cells per well in 24-well-plates) were transfected with anti-p23 or control siRNAs (ON-TARGET plus SMART pool L-004496-00 and ON-TARGET plus Non targeting pool, Dharmacon) at a final concentration of 25 nM using the DharmaFECT reagent (Dharmacon) according to the manufacturer's recommendations. For viral minigenome assays, plasmids pcDNA3.1-PB1, -PB2, -PA and -NP (0.25, 0.25, 0.25, 0.5 µg) were transfected together with the pPR7-FluA-Luc plasmid (0.1 µg) using 5 µl of the Fugene-HD transfection reagent (Roche). At 24 hours post-transfection, cell lysates were prepared and luciferase activity was measured, using the Lysis Buffer and substrate provided in the *Renilla* Luciferase Assay System kit (Promega) and a Tecan luminometer (Berthold).

For GR-mediated gene transactivation assays, subconfluent monolayers of 293T cells (4×10^5^ cells per well in 12-well-plates) were pre-incubated for 16 hours in serum free medium prior to cotransfection with a GR expression vector, a GR-*Firefly* luciferase reporter plasmid and a pTK-*Renilla* luciferase expressing vector (0.5 µg of each plasmid) using 5 µL of the FuGENE HD reagent (Roche). At 24 hours post-transfection cells were infected at a m.o.i. of 5 pfu/cell with the A/WSN/33 virus. After one hour of adsorption, the viral suspension was replaced with medium supplemented with dexamethasone and cells were incubated at 35°C for 10 hours. Cell lysates were prepared and luciferase activities were measured using Luciferase Assay kits (Promega).

### Indirect immunofluorescence assay

293T cells on coverslips were transfected using the FuGENE HD reagent (Roche) with a PB2-flag expression vector alone or in combination with PB1 and PA expression vectors. At 24 h post-transfection cells were fixed with PBS-4% paraformaldehyde for 20 min and permeabilized with PBS-0.1% Triton X100 for 15 min. They were incubated with a mixture of the mouse monoclonal anti-p23 (Abcam, diluted 1/250) and the rabbit polyclonal anti-Flag (Sigma, diluted 1/400) antibodies, and then with a mixture of AF555-coupled anti-mouse IgG and AF488 coupled anti-rabbit IgG secondary antibodies (Invitrogen, diluted 1/1000).

A549 or 293T cells on coverslips were infected with the PB2-HA recombinant virus at a m.o.i. of 5 pfu/cell or mock-infected, and incubated at 37°C. At 4–8 hours post-infection, cells were fixed and permeabilized as indicated above. They were incubated with a mixture of the mouse monoclonal anti-p23 and the rabbit polyclonal anti-Hsp90 (Santa Cruz Biotechnology, diluted 1/100) antibodies, and then with a mixture of AF640-coupled anti-mouse IgG secondary antibody (Invitrogen, diluted 1/500), AF555-coupled anti-rabbit IgG secondary antibody (Invitrogen, diluted 1/500), and AF488-coupled anti-HA antibody (Invitrogen, diluted 1/200). Alternatively, cells were incubated with dexamethasone 10 nM for 1 hour at 37°C prior to fixation and permeabilization. They were incubated with a mixture of the rat anti-HA (Roche, diluted 1/1,000) and mouse anti-glucocorticoid receptor (Affinity BioReagents, diluted 1/300) monoclonal antibodies, and then with a mixture of AF555-coupled anti-mouse IgG secondary antibody (Invitrogen, diluted 1/1,000) and AF488-coupled anti-rat IgG secondary antibody (Invitrogen, diluted 1/500). The samples were analyzed under a fluorescence microscope (Zeiss Axioplan 2 imaging- Zeiss ApoTome).

### Western-blot assays

For western blot assays, cells in 35-mm dishes were resuspended directly in 300 µl of sample loading buffer. The cell lysates were centrifuged for 2 mn at 16,000 g on a QIAShredder column (QIAGEN), heated for 3 min at 95°C and analyzed by electrophoresis on a 4–12% Bis-Tris NuPAGE gel (Invitrogen) and western blotting using PVDF membranes. The membranes were incubated overnight at 4°C with primary antibodies directed against p23 (Abcam, diluted 1/1,000), GST (Upstate Cell Signalling Solutions, diluted 1/4,000), Hsp90 (Santa Cruz Biotechnology, diluted 1/1,000) or Histone3 (ab1791, Abcam, diluted 1/3,000), or with rabbit polyclonal serum directed against the PB1, PB2, or PA proteins (kindly provided by J. Ortin, Centro Nacional de Biotecnologia, Madrid, Spain, diluted 1/5,000) or against A/PR/8/34 virions ([22], diluted 1/10,000) in PBS with 1% BSA, 0.25% Tween20. Membranes were then incubated for 1 h at room temperature with peroxydase-conjugated secondary antibodies, with the ECL+ substrate (GE Healthcare), and scanned for chemiluminescence using a G-Box (Syngene).

## Results

### p23 associates with influenza virus polymerase

In order to identify cellular proteins associated to influenza virus RNPs, we produced a recombinant A/WSN/33 (WSN) influenza A virus expressing a PB2 protein fused to a Strep-tag epitope at the C-terminus (PB2-Strep virus). The Strep-tag is a short polypeptide which binds specifically to Strep-tactin, a derivative of streptavidin [23]. The packaging signal overlapping the coding and non-coding regions at the 5′ end of the PB2 segment was conserved by duplicating the 109 last nucleotides encoding PB2 between the Strep-tag sequence and the 5′NCR, as described previously [24]. The resulting PB2-Strep virus replicated efficiently, and showed no genetic instability upon sequential amplifications on MDCK cells (data not shown).

**Figure 1:**
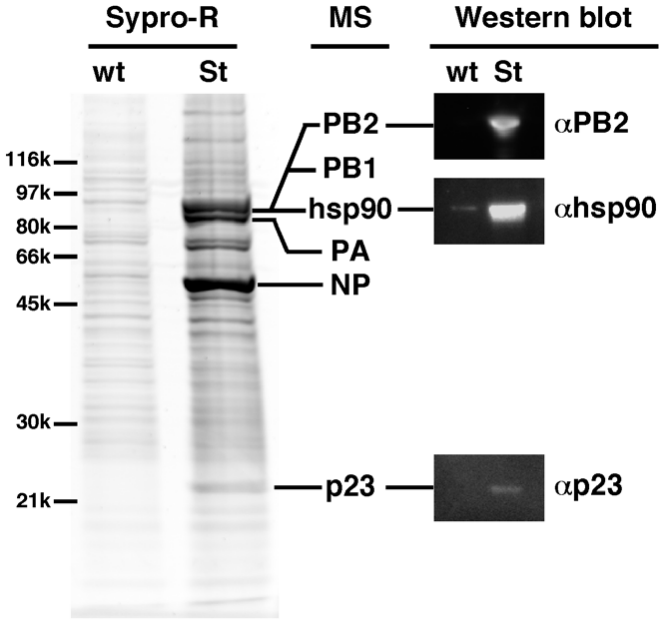
Co-purification of influenza virus polymerase and p23 upon infection of 293T cells with a PB2-Strep-WSN virus. 293T cells were infected with the WSN (WT) or WSN-PB2-Strep (Strep) virus at a m.o.i. of 5 for 6 hours. Cell lysates were loaded onto a Strep-tactin column, and bound proteins were eluted in desthiobiotine elution buffer. Eluted proteins were analysed on a 4–15% polyacrylamide gel and stained with Sypro-Ruby. Proteins from individual gel slices were analysed by mass spectrometry (MS). For confirmation, western blot analysis of eluted proteins was performed using antibodies specific for the PB2, Hsp90 and p23 proteins.

The PB2-Strep and wild-type (PB2-wt) viruses were used in parallel to infect 4×10^9^ 293T cells at a multiplicity of infection (m.o.i.) of 5 pfu/cell. Cytoplasmic extracts were prepared at 6 hours post-infection (hpi), and were loaded onto Strep-tactin columns. Native PB2-Strep complexes were eluted using desthiobiotin, and subjected to SDS-PAGE analysis. As shown in Figure 1, Sypro-Ruby staining of the gel revealed a number of bands which were present in the sample derived from cells infected with the PB2-Strep virus, but not from cells infected with the PB2-wt virus. Slices of the gel were sent to Institut Pasteur Proteomics Facility. As expected, slices corresponding to the major bands corresponded to the PB2, PB1, PA and NP viral proteins (Figure 1), which was in agreement with our previous observations using PB2-Strep viruses [24], [25]. The slice containing PB2 and PB1 was also found to contain the Hsp90 protein, whereas a band of lower molecular weight corresponded to the p23 co-chaperone of Hsp90. Western-blot analysis using anti-Hsp90 and anti-p23 antibodies confirmed that Hsp90 and p23 were specifically co-purified with PB2-Strep complexes (Figure 1).

**Figure 2:**
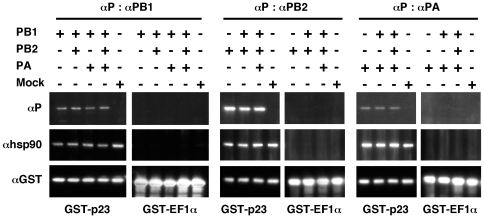
Co-purification of a GST-p23 fusion protein and the influenza virus polymerase subunits upon co-expression in 293T cells. 293T cells were co-transfected with expression plasmids for GST-p23 or GST-EF1α fusion proteins, together with expression plasmids for the WSN-PB1, WSN-PB2, and/or WSN-PA proteins. At 48 hours post-transfection, cell lysates were incubated with Glutathion Sepharose beads overnight, and bound proteins were eluted in Laemmli buffer. Eluted proteins were analysed by western blot using antibodies specific for the GST, Hsp90, p23, PB1, PB2 and PA proteins.

Co-purification assays were then performed using a GST-p23 fusion protein transiently expressed in 293T cells. A pCMV-GST-p23 expression vector was transfected in 293T cells together with pcDNA3.1 expression vectors for the WSN-PB1, -PB2 or -PA proteins, separately or in combination. Similar to p23, EF1-α is an abundant, constitutively expressed, cytoplasmic protein, and therefore a GST-EF1α construct was used in parallel as a control. At 48 h post-transfection, total cell extracts were prepared and incubated with glutathion beads as described under the Materials and Methods section. The GST-p23 complexes were washed, eluted using Laemmli buffer and subjected to western-blot analysis. As expected the Hsp90 protein was co-purified with the GST-p23 protein, but not with the GST-EF1α protein (Figure 2). Each of the PB1, PB2 and PA subunits of the polymerase complex bound specifically to the GST-p23 protein, whether expressed alone, or in combination with one or both of the other subunits (Figure 2).

### Viral replication is not impaired in p23-deficient cultured cells

**Figure 3:**
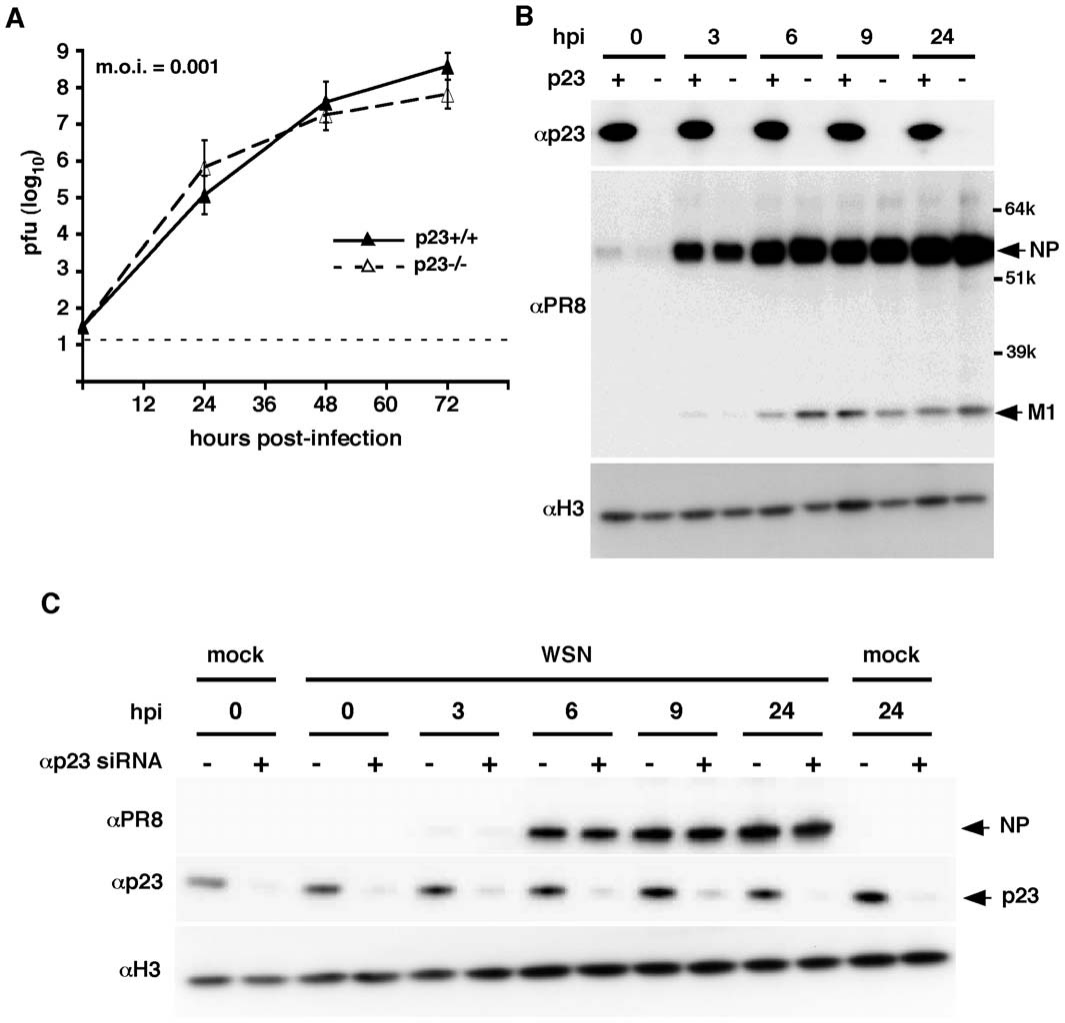
Influenza virus replication on p23^−/−^ and wild-type mouse embyonic fibroblasts. **A.** Subconfluent monolayers of p23^−/−^ and wild-type mouse embryonic fibroblasts (MEFs) were infected at a m.o.i. of 10^−3^ pfu/cell with the WSN virus and incubated for 72 h at 37°C. At the indicated time-points, supernatants were harvested and viral titers were determined by plaque assays on MDCK cells. The mean values ± SD from 3 independent experiments are shown. The horizontal dotted line represents the limit of detection in the plaque assays. **B.** Subconfluent monolayers of p23^−/−^ and wild-type MEFs were infected at a m.o.i. of 10 pfu/cell with the WSN virus and incubated for 24 h at 37°C. Total cell lysates prepared at the indicated time-points were analysed by western blot, using an anti-p23 antibody, a polyclonal serum directed against the A/PR/8/34 virus which enables detection of the NP and M1 proteins of WSN, and a polyclonal anti-Histone3 antibody. Data are representative of two independent experiments. **C.** 293T cells were transfected with anti-p23 or control siRNAs. At 48 hours post-transfection, they were infected at a m.o.i. of 10 pfu/cell with the WSN virus and incubated for 24 h at 37°C. Total cell lysates prepared at the indicated time-points were analysed by western blot, as in **B**.

To investigate the functional relevance of p23 interaction with the influenza polymerase complex in the replication cycle of influenza viruses, we compared viral growth on mouse embryonic fibroblasts either derived from p23-deficient mice (p23^−/−^) or from control wild-type mice (wt) [21]. Following infection at a m.o.i. of 10^−3^ pfu/cell, viral titers in the culture supernatants were determined at different times post-infection by plaque assay on MDCK cells. The WSN virus replicated at a similar rate on p23^−/−^ and wt cells, titers of 10^7^–10^8^ pfu/ml being observed at 48 hpi (Figure 3A). The same observations were made when the cells were incubated at 33°C or 39°C instead of 35°C upon infection, or when a recombinant virus expressing the PB1, PB2, PA and NP proteins derived from the A/Paris/908/97 (H3N2) human isolate was used instead of the A/WSN/33 virus (data not shown).

To further document the effect of p23 depletion on influenza virus replication, we performed single-cycle growth assays on p23^−/−^ and wt cells. Following infection with the WSN virus at a high m.o.i. of 10 pfu/cell, total cell extracts were prepared at various times post-infection and analyzed by western-blot using a polyclonal serum to detect the viral NP and M1 viral proteins, or cells were fixed and analyzed by immunofluorescence to detect the PB2 protein. As shown in Figure 3B the NP and M1 proteins steady-state levels were similar in p23^−/−^ and wt cells. The nuclear accumulation of the PB2 viral polymerase subunit was not delayed in p23^−/−^ cells (Figure S1). The viral titers in the supernatants of p23^−/−^ and wt cells were in the same range, and reached 10^6^–10^7^ pfu/ml at 48 hpi (data not shown).

Single-cycle growth assays were also performed in 293T cells transiently transfected with anti-p23 siRNAs. The steady-state level of p23, as evaluated by western-blot analysis of serial dilutions of total cell extracts, showed a significant reduction in 293T transfected with the anti-p23 siRNAs as compared to the control siRNAs (Figure 3C, middle panel). Following infection with the WSN virus at a high m.o.i. of 10 pfu/cell, accumulation of the NP and M1 proteins occurred at the same rate in anti-p23 and control siRNA-treated cells (Figure 3C, upper panel).

The viral polymerase activity was assayed in anti-p23 and control siRNA-treated 293T cells, by co-expressing transiently the PB1, PB2, PA and NP proteins of WSN together with an influenza-like RNA containing the luciferase reporter gene. The efficiency with which the influenza-like RNA underwent transcription/replication, as monitored by the levels of luciferase activity in transfected cell extracts, was similar in both types of cells (data not shown).

### p23 relocalizes to the nucleus in influenza virus-infected cells

**Figure 4:**
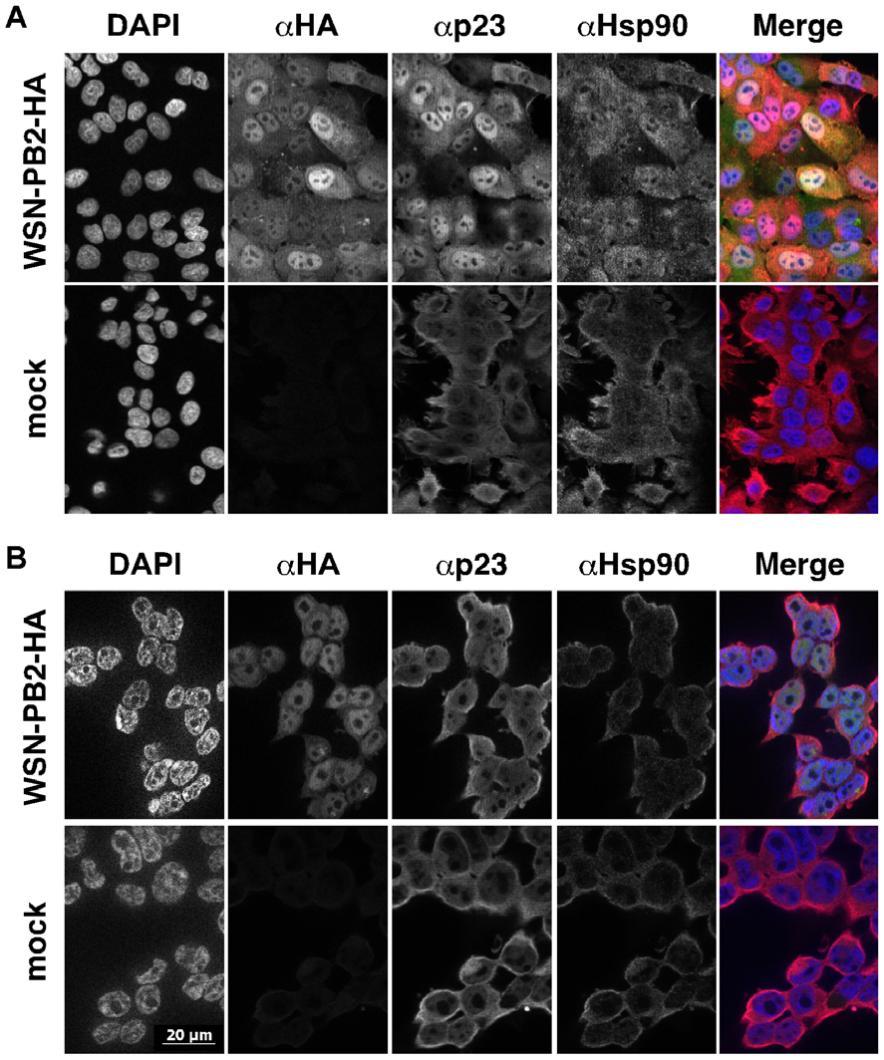
Relocalization of p23 to the nucleus in influenza virus-infected cells. A549 (**A**) or 293T (**B**) cells were infected at a m.o.i. of 10 pfu/cell with the WSN-PB2-HA virus (upper panels) or mock-infected (lower panels). At 8 hpi (**A**) or 6 hpi (**B**), cells were fixed, permeabilized, and stained with antibodies specific for the HA tag (PB2), and for the p23 and the Hsp90 proteins. Samples were analyzed under a fluorescence microscope (Zeiss Axioplan 2 Imaging - Zeiss ApoTome). A merge of the signals corresponding to DAPI (blue), HA (green) and p23 (red) is shown. Data are representative of three independent experiments.

The subcellular localization of p23 in influenza virus- and mock-infected A549 and 293T cells was compared. A recombinant WSN virus expressing a PB2 protein fused with the HA tag at the C-terminus was used in these experiments, to allow simultaneous labeling of the viral PB2 and cellular p23 and Hsp90 proteins. The anti-HA antibody did not recognize the WSN hemagglutinin, as expected from the fact that the YPYDVPDY sequence of the HA tag is not present in this hemagglutinin, and as demonstrated by the background levels of fluorescence measured on control cells infected with the wild-type WSN virus (Figure S2). In uninfected A549 and 293T cells, p23 and Hsp90 were predominantly detected in the cytoplasm (Figure 4A and 4B, respectively, lower panels). In infected cells, relocalisation of p23 to the nucleus became detectable at 4 hpi (data not shown) and was very obvious at 6–8 hpi (Figure 4A and 4B, upper panels). Relocalisation of Hsp90 was also observed, in agreement with previously published data [5], but was less pronounced as compared to p23 relocalisation (Figure 4A and 4B, upper panels).

**Figure 5:**
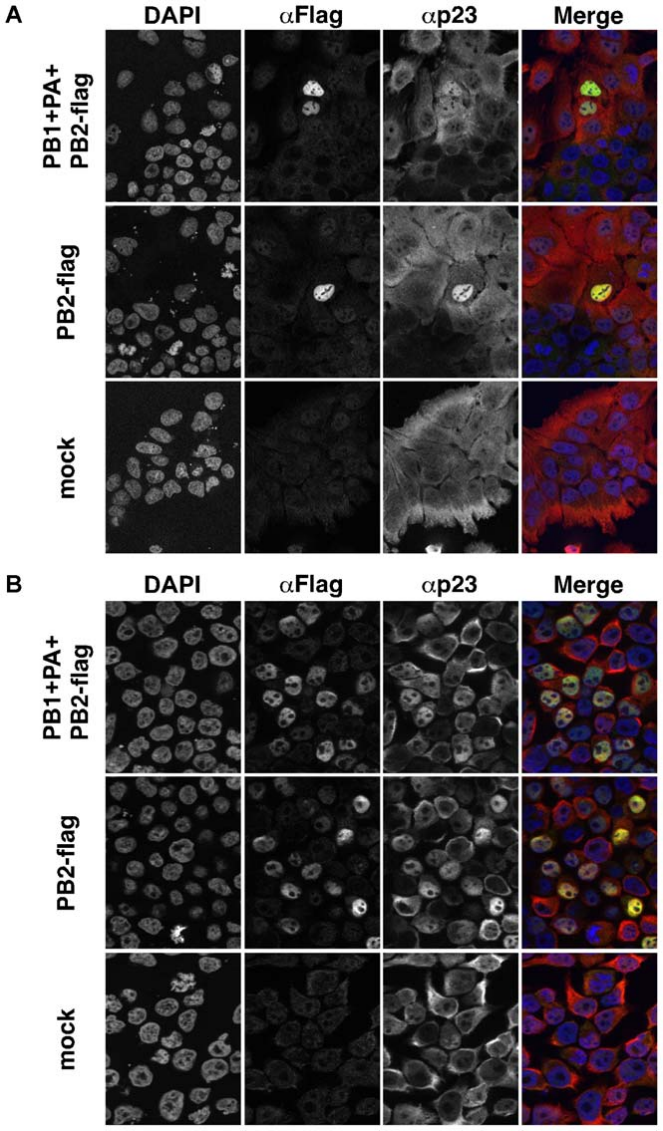
Relocalization of p23 to the nucleus of cells transiently expressing influenza virus polymerase subunits. A549 (**A**) or 293T (**B**) cells were transfected with a WSN-PB2-Flag expression plasmid alone or in combination with PB1 and PA expression plasmids, or mock-transfected. At 24 hours post-transfection, cells were fixed, permeabilized, and stained with antibodies specific for the Flag tag (PB2) and for the p23 protein. Samples were analyzed under a fluorescence microscope (Zeiss Axioplan 2 Imaging - Zeiss ApoTome). A merge of the signals corresponding to DAPI (blue), Flag (green) and p23 (red) is shown. Data are representative of two independent experiments.

The subcellular localization of p23 was then examined in transfected cells transiently expressing the viral polymerase subunits. Relocalisation of p23 to the nucleus was clearly observed in A549 or 293T cells transiently expressing the PB2 subunit, either alone or in combination with the PB1 and PA subunits (Figure 5A and 5B, respectively). Nuclear relocalisation of p23 was also observed in 293T cells expressing the PA subunit alone but not in cells expressing the PB1 subunit alone (data not shown), which was consistent with the fact that unlike PA, PB1 is not efficiently imported in the nucleus when expressed on its own [26]. Nuclear relocalisation of p23 was not observed in cells expressing the viral nucleoprotein, although the nucleoprotein was detected in the nucleus as well as in the cytoplasm of transfected cells (Figure S3). Overall, these observations supported the hypothesis that the specific association of p23 with the viral polymerase subunits is driving p23 relocalisation to the nucleus in infected cells.

### Glucocorticoid-receptor mediated signalling is impaired in influenza virus infected cells

**Figure 6:**
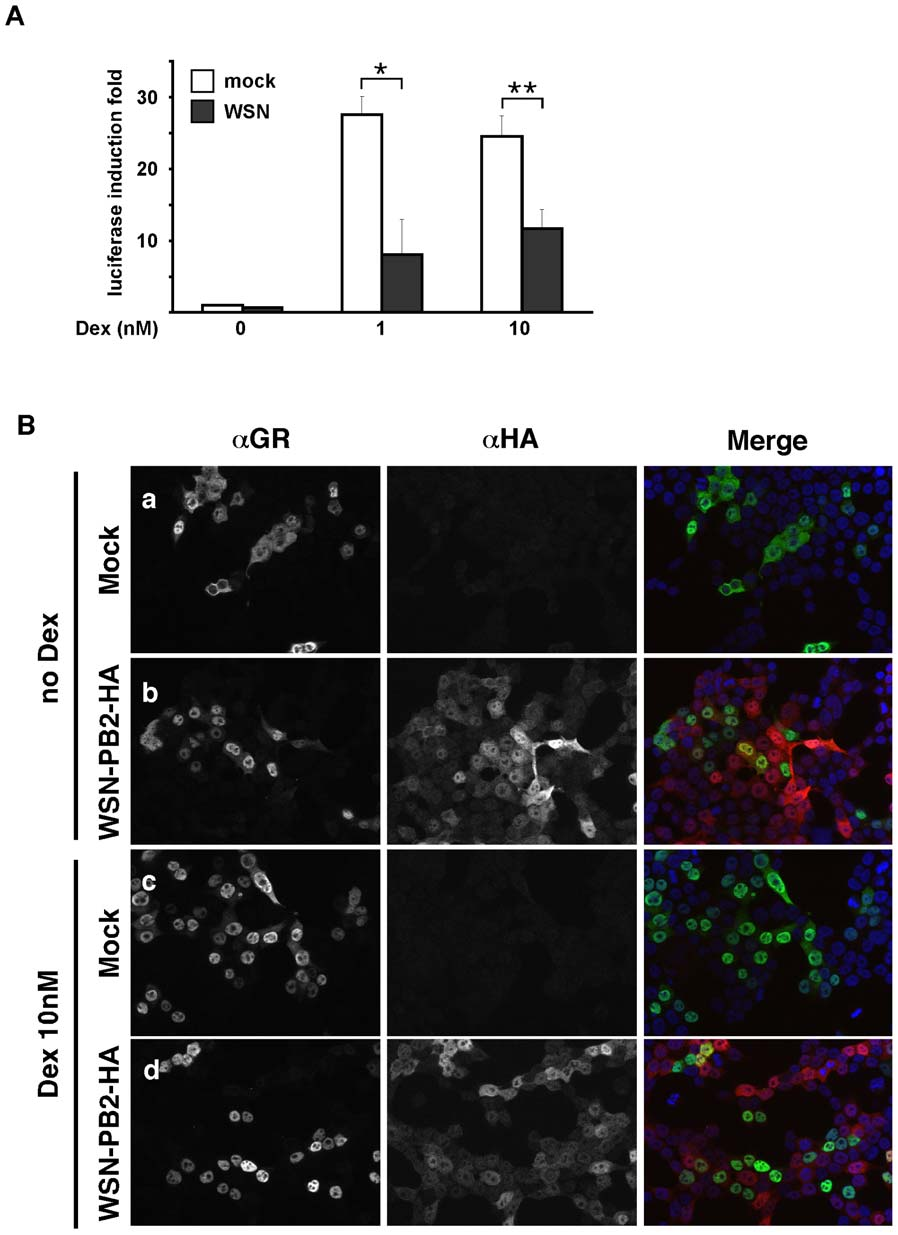
Altered glucocorticoid receptor-mediated transcriptional response and glucocorticoid receptor localization in influenza virus-infected cells. **A.** 293T cells were co-transfected with a GR expressing plasmid, a reporter plasmid containing the *Firefly*-luciferase gene under the control of GR-responsive elements, and a *Renilla*-luciferase expressing plasmid. At 48 hours post-transfection, cells were infected with the WSN virus at a m.o.i. of 5 pfu/ml (grey bars) or mock-infected (white bars), and incubated for 10 hours in the presence of dexamethasone at the indicated concentrations. *Firefly*-luciferase activities were measured in cell lysates and normalized with respect to *Renilla*-luciferase activities. The results are expressed as the mean ± SD of four independent experiments performed in duplicate, and as fold induction of *Firefly*-luciferase activity relative to the untreated uninfected control. * p<0.05; ** : p<0.02 (Student's *t* test). **B.** 293T cells were transfected with a GR expressing plasmid or mock-transfected. At 24 hours post-transfection, cells were infected with the WSN-PB2-HA virus at a m.o.i. of 5 pfu/ml (panels b and d) or mock-infected (panels a and c). At 5 hpi, they were incubated for one hour in the absence (panels a and b) or in the presence of 10 nM dexamethasone (panels c and d). The cells were then fixed, permeabilized, and stained with antibodies specific for the HA tag (PB2) and for the GR. Samples were analyzed under a fluorescence microscope (Zeiss Axioplan 2 Imaging - Zeiss ApoTome). A merge of the signals corresponding to DAPI (blue), HA (red) and GR (green) is shown. Data are representative of two independent experiments.

As a component of the Hsp90 chaperone complex, p23 plays a complex role in the maturation and function of the glucocorticoid receptor (GR) (reviewed in [16]). In p23-deficient cells, the GR-mediated gene transactivation is impaired, which coincides with a delayed nuclear translocation of the GR in response to dexamethasone [21]. We hypothetized that the strong relocalisation of p23 observed in influenza-virus infected cells might have an impact on GR signalling. To evaluate GR-mediated gene transactivation in influenza-infected *vs* mock-infected cells, 293T cells were cotransfected with a GR expressing plasmid, a reporter plasmid containing the *Firefly*-luciferase gene under the control of a minimal TK promoter and GR-responsive elements, and a *Renilla*-luciferase expressing construct. At 48 hours post-transfection, they were infected with WSN at a m.o.i. of 5 pfu/ml, or mock-infected. After one hour of adsorption, the viral inoculum was removed, and replaced with fresh medium either supplemented with dexamethasone or not. After 10 hours of stimulation, cell lysates were prepared and *Firefly*-luciferase activities were determined and normalized with respect to *Renilla*-luciferase activities. As shown in Figure 6A, the dexamethasone-induced transactivation of the *Firefly*-luciferase gene in infected cells (12- and 19-fold at 1 nM and 10 nM dexamethasone, respectively) was reduced as compared to in mock-infected cells (28- and 25-fold at 1 nM and 10 nM dexamethasone, p<0.02 and p<0.05, respectively). The subcellular localisation of the GR was examined in WSN-PB2-HA- and in mock-infected 293T cells, upon incubation in the absence or in the presence of 10 nM dexamethasone. An indirect immunoassay was performed at 5 hpi, using a mixture of an anti-HA and an anti-GR antibody. Uninfected cells generally showed cytoplasmic or cytoplasmic and nuclear GR staining in the absence of dexamethasone (Figure 6B, panel a), and predominantly nuclear GR upon stimulation with dexamethasone (Figure 6B, panel c). Strikingly, infected cells showed nuclear relocalisation of the GR in the absence as well as in the presence of dexamethasone (Figure 6B, panels b and d, respectively). Sequestration of the GR in the nucleus in its hormone-free conformation could contribute to the impairment of GR-mediated signalling by reducing the pool of cytoplasmic GR available for hormone recognition.

**Figure 7:**
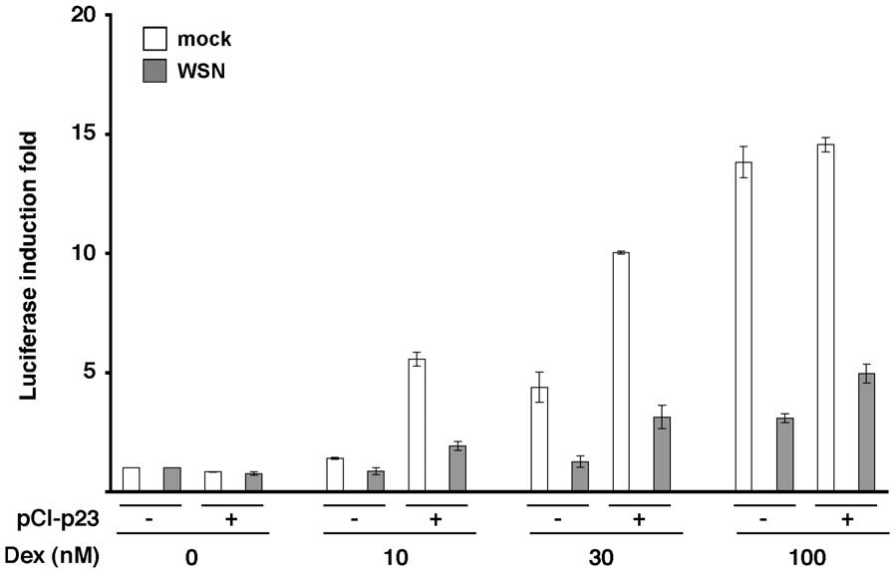
Altered glucocorticoid receptor-mediated transcriptional response in influenza virus-infected p23^−/−^ mouse embyonic fibroblasts. p23^−/−^ MEFs were co-transfected with a GR expressing plasmid, a reporter plasmid containing the *Firefly*-luciferase gene under the control of GR-responsive elements, and a *Renilla*-luciferase expressing plasmid, together with the pCI-p23 plasmid (+) or the control pCI plasmid (−). At 24 hours post-transfection, cells were infected with the WSN virus at a m.o.i. of 5 pfu/ml (grey bars) or mock-infected (white bars), and incubated for 10 hours in the presence of dexamethasone at the indicated concentrations. *Firefly*-luciferase activities were measured in cell lysates and normalized with respect to *Renilla*-luciferase activities. The results are expressed as the mean ± SD of duplicate assays, and are representative of 2 independent experiments performed in duplicates.

We finally asked whether the observed reduction of GR-mediated gene transactivation in influenza-infected *vs* mock-infected cells was mediated by p23. To this end, the luciferase reporter assay described above was repeated using p23^−/−^ MEFs, either transfected with a p23 expression plasmid or mock-transfected. Upon infection, cells were incubated in the presence of 10 to 100 nM dexamethasone. In mock-infected cells, transactivation of the *Firefly*-luciferase gene increased with the dose of dexamethasone and was stronger in the presence of the p23 expression plasmid (Figure 7, white bars), which was in agreement with the known functions of p23 in GR-mediated signalling [21], [27], [28], [29]. A negative effect of viral infection on transactivation of the *Firefly*-luciferase gene in response to dexamethasone was clearly observed, in the presence as well as in the absence of the p23 expression plasmid (Figure 7, grey bars). These data indicated that the observed viral effect could occur through a p23-independent pathway.

## Discussion

We used a recombinant influenza virus expressing a PB2 protein fused to a Strep-tag for the co-purification and identification of interacting host factors during the course of viral infection. The Strep-tag has been described as an appropriate tag to purify protein complexes from crude extracts under mild elution conditions and with good yields [23]. Indeed, PB2-Strep viruses allowed co-purification of PB2 and PB2-associated viral proteins such as PB1, PA and NP (this study, and [24]), as well as a number of cellular proteins that have already been identified as cellular interactors of the viral polymerase or nucleoprotein, such as the RNA polymerase II large subunit [10], [24], Hsp90 (this study), Hsp70, and actin (data not shown). The PB2-Strep virus is potentially a useful tool to explore the spatial and temporal dynamics of these protein interactions.

We found that the p23 cochaperone of Hsp90 was associated with PB2 in cytoplasmic extracts from 293T cells infected with the WSN-PB2-Strep virus. A transient co-expression/purification assay confirmed that a GST-p23 fusion protein, but not a control GST-EF1α fusion protein, bound to each of the PB1, PB2 and PA polymerase subunits. In both experimental systems, the elution samples containing the p23 and polymerase subunit(s) also contained the Hsp90 chaperone, in agreement with existing data showing PB2-Hsp90 and p23-Hsp90 interactions [5], [16]. Whether p23 is directly or indirectly binding to influenza polymerase subunits thus remains unclear. p23 was found in complexes with Hsp90 and a variety of client proteins, including hepatitis B virus polymerase [30], but whether p23 is able to interact directly with these proteins has not been established. Since Hsp90 does not bind efficiently to PA [5, Chase and Schwemmle, unpublished observations], the p23-PA association suggests that p23 can associate with the influenza polymerase, at least partially, independently of Hsp90. We observed relocalization of p23 in the nucleus of infected cells, which is consistent with the biochemical interaction data as the viral polymerase accumulates in the nuclear compartment. Nuclear retention was more pronounced for p23 than for Hsp90, which might be indicative of some Hsp90-independent interaction between p23 and influenza polymerase occuring in the nucleus. Relocalization of p23 in the nucleus was also observed in cells that transiently co-expressed the three viral polymerase subunits as well as in cells that expressed the PB2 or PA subunit alone, but not in cells that expressed the viral NP. These observations further demonstrated that the interaction between p23 and influenza virus polymerase was specific and did not require the presence of other viral proteins. Nuclear sequestration of p23 may alter some of p23 and Hsp90 functions in influenza virus-infected cells.

Whereas Hsp90 is thought to play a role in the import and assembly of newly synthetized influenza polymerase subunits [5], [31], we show here that p23 depletion has no impact on viral multiplication efficiency in cultured murine embryonic fibroblasts or 293T cells. The chaperoning activity of Hsp90 is regulated by ATP and by a large variety of cofactors, which control Hsp90's shuttling between open and closed conformations [32]. p23 is considered a general cochaperone of Hsp90 client proteins which acts by stabilizing the ATP-bound closed conformation of Hsp90 [33], [34]. However, p23 was found to be an enhancing but not an essential factor for several Hsp90 client proteins, and might be dispensable for some of them [16]. Functional redundancy beween p23 and other cochaperone(s) of Hsp90 cannot be excluded [35], and could also account for our observations.

The usually observed nucleo-cytoplasmic shuttling of p23 is related to its complex role in glucocorticoid signalling. In the absence of hormone, glucocorticoid receptors (GRs) are mainly located in the cytoplasm, and p23 promotes their folding into a hormone-binding conformation by stabilizing GR-Hsp90-p60-Hsp70 complexes [27]. Upon hormone binding, GRs rapidly accumulate in the nucleus and upregulates the transcription of a large array of genes, such as IκBα or GC-induced leucine zipper, which are thought to be involved in the GC-mediated anti-inflammatory effects [36]. Nuclear translocation of the GR in response to hormonal stimulation is delayed in p23-deficient cells [21], and recent data suggest that the p23 and p60 co-chaperones play a role in the nuclear import of GRs [28]. Furthermore, p23 acts independently of Hsp90 in the nucleus by mediating the disassembly of transcriptional regulatory complexes formed by intra-nuclear hormone-bound GRs [37].

This complex regulatory function of p23 at various steps of the GR signalling pathway is likely to be disrupted by the strong relocalisation of p23 in the nucleus of influenza-virus infected cells. Indeed, we found that the GR sub-cellular localisation is altered and the GR-mediated gene transactivation is impaired in influenza virus-infected cells. Viral infection still shows a negative effect on GR-mediated gene transactivation in cells that are defective for p23. This observation, although it does not rule out a contribution of p23, indicates that viral-induced inhibition of GR-mediated signalling can occur through a p23-independent and redundant pathway. A possible approach to unravel the contribution of p23 would be to identify influenza variants defective for p23 binding and to compare their impact on GR signalling.

Our observation of an impaired response to glucocorticoids in influenza infected cells adds to few previously published data showing alterations of the GR activity upon viral infections (reviewed in [38]). A recent study reports repression of GR-mediated gene transactivation upon infection with the Respiratory Syncitial Virus [39]. Very little is known about the interaction of influenza virus and the GR signalling pathway. In vitro, influenza viruses are usually grown in the absence of serum and thus in the absence of glucocorticoids. Any potential downstream effects of an altered GR activity upon influenza infection have therefore most likely been overlooked. Influenza virus infection in mice was shown to result in a sustained increase in serum glucocorticoid levels [40] and a transcriptional induction of two GR-regulated genes, metallothionein I (MT-I) and II (Mt-II) [41]. Our data suggest that, besides the systemic activation of the hypothalamic-pituitary-adrenal axis with resulting release of glucocorticoids, the intensity of the glucocorticoid response to influenza infection could be limited by intracellular viral-induced mechanisms. A better understanding of these mechanisms may have implications for the therapeutic treatment of severe cases of influenza in which the balance between pro-inflammatory and anti-inflammatory cytokines is a major physiopathological parameter [42].

## Supporting Information

Figure S1 **Subcellular localisation of the PB2 protein upon influenza virus infection of p23^−/−^ and wild-type mouse embyonic fibroblasts.** p23^−/−^ and wild-type p23^+/+^ mouse embryonic fibroblasts (MEFs) were infected at a m.o.i. of 10 pfu/cell with the WSN-PB2-HA virus. At 5 hpi, cells were fixed, permeabilized and stained with antibodies specific for the HA tag (PB2) and for the Hsp90 protein. Samples were analyzed under a fluorescence microscope (Zeiss Axioplan 2 Imaging - Zeiss ApoTome). A merge of the signals corresponding to DAPI (blue), HA (red) and Hsp90 (green) is shown.
(TIF)

Figure S2 **Specific recognition of the HA-tag and not the WSN virus hemagglutinin by the anti-HA antibody.** A549 (**A**) or 293T (**B**) cells were infected at a m.o.i. of 10 pfu/cell with the WSN-PB2-HA virus (upper panels) or the WSN wild-type virus (lower panels). At 8 hpi (**A**) or 6 hpi (**B**), cells were fixed, permeabilized, and stained with antibodies specific for the HA tag (PB2) and for the p23 protein. Samples were analyzed under a fluorescence microscope (Zeiss Axioplan 2 Imaging - Zeiss ApoTome). A merge of the signals corresponding to DAPI (blue), HA (green) and p23 (red) is shown.
(TIF)

Figure S3 **Subcellular localisation of p23 in 293T cells transiently expressing the viral PB2 or NP protein.** 293T cells were transfected with a plasmid encoding NP (middle pannel), PB2-Flag (lower panel) or mock-transfected (upper panel). At 24 hpi cells were fixed, permeabilized, and stained with antibodies specific for the Flag tag (PB2) or the NP protein, together with an anti-p23 antibody. Samples were analyzed under a fluorescence microscope (Zeiss Axioplan 2 Imaging - Zeiss ApoTome). A merge of the signals corresponding to DAPI (blue), Flag or NP (green) and p23 (red) is shown.
(TIF)
