# Supplementary figures and images for: Influenza Virus Infection Induces the Nuclear Relocalization of the Hsp90 Co-Chaperone p23 and Inhibits the Glucocorticoid Receptor Response
Source: PLoS One. 2011 Aug 10;6(8):e23368. doi: 10.1371/journal.pone.0023368 (PMC3154441; doi:10.1371/journal.pone.0023368)

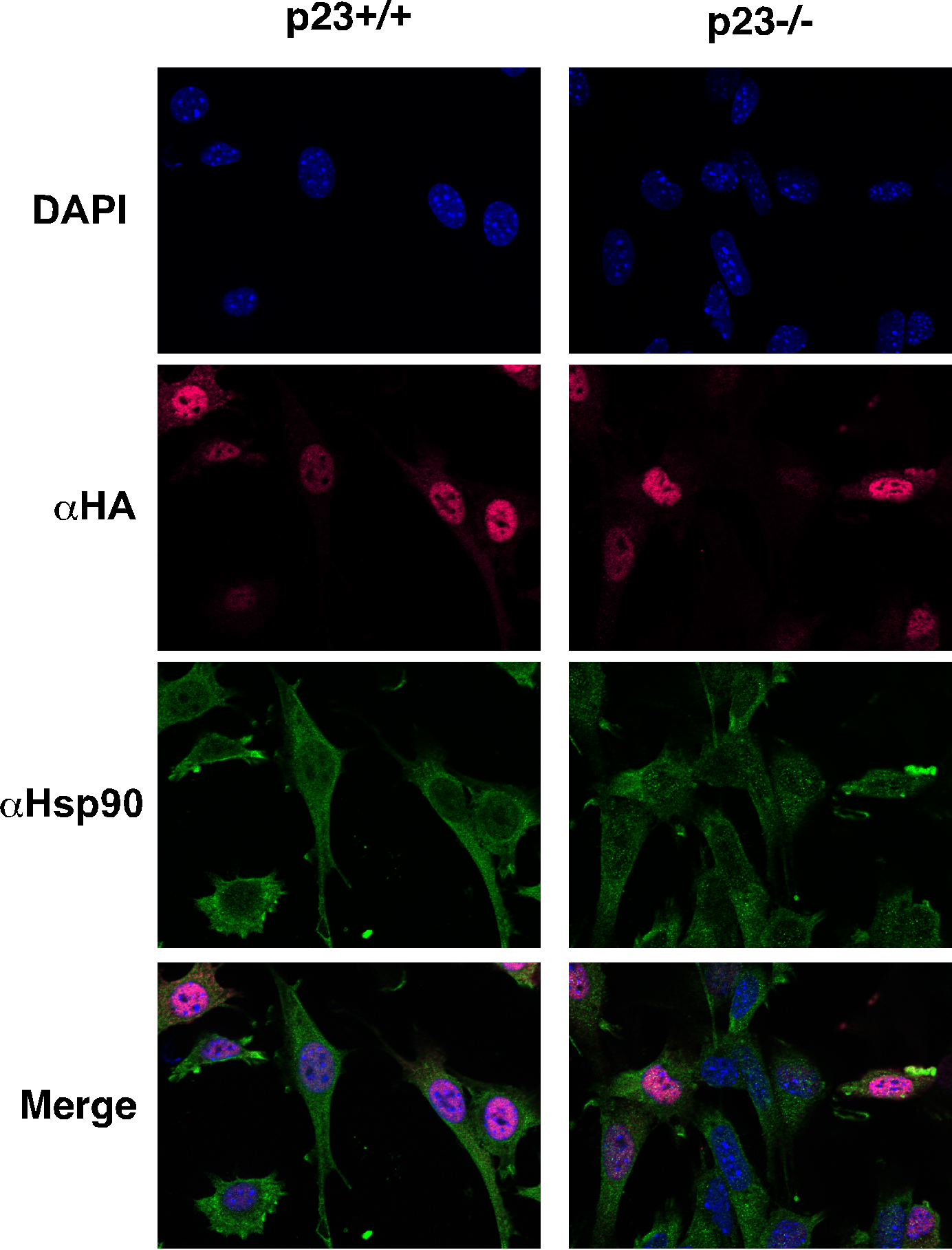

Supplement: Figure S1 — Subcellular localisation of the PB2 protein upon influenza virus infection of p23−/− and wild-type mouse embyonic fibroblasts. p23−/− and wild-type p23+/+ mouse embryonic fibroblasts (MEFs) were infected at a m.o.i. of 10 pfu/cell with the WSN-PB2-HA virus. At 5 hpi, cells were fixed, permeabilized and stained with antibodies specific for the HA tag (PB2) and for the Hsp90 protein. Samples were analyzed under a fluorescence microscope (Zeiss Axioplan 2 Imaging - Zeiss ApoTome). A merge of the signals corresponding to DAPI (blue), HA (red) and Hsp90 (green) is shown. (TIF) [file pone.0023368.s001.tif]

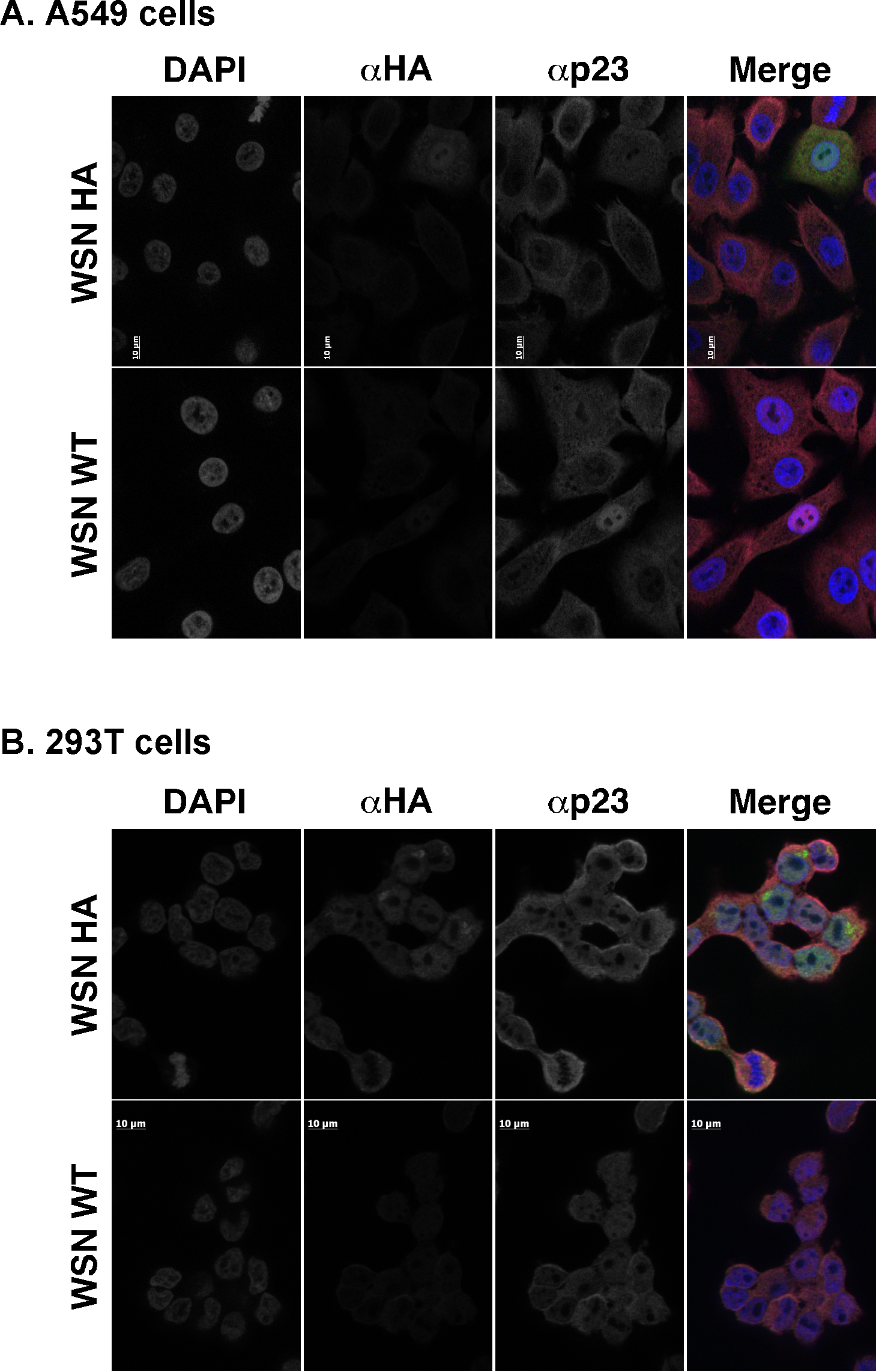

Supplement: Figure S2 — Specific recognition of the HA-tag and not the WSN virus hemagglutinin by the anti-HA antibody. A549 (A) or 293T (B) cells were infected at a m.o.i. of 10 pfu/cell with the WSN-PB2-HA virus (upper panels) or the WSN wild-type virus (lower panels). At 8 hpi (A) or 6 hpi (B), cells were fixed, permeabilized, and stained with antibodies specific for the HA tag (PB2) and for the p23 protein. Samples were analyzed under a fluorescence microscope (Zeiss Axioplan 2 Imaging - Zeiss ApoTome). A merge of the signals corresponding to DAPI (blue), HA (green) and p23 (red) is shown. (TIF) [file pone.0023368.s002.tif]

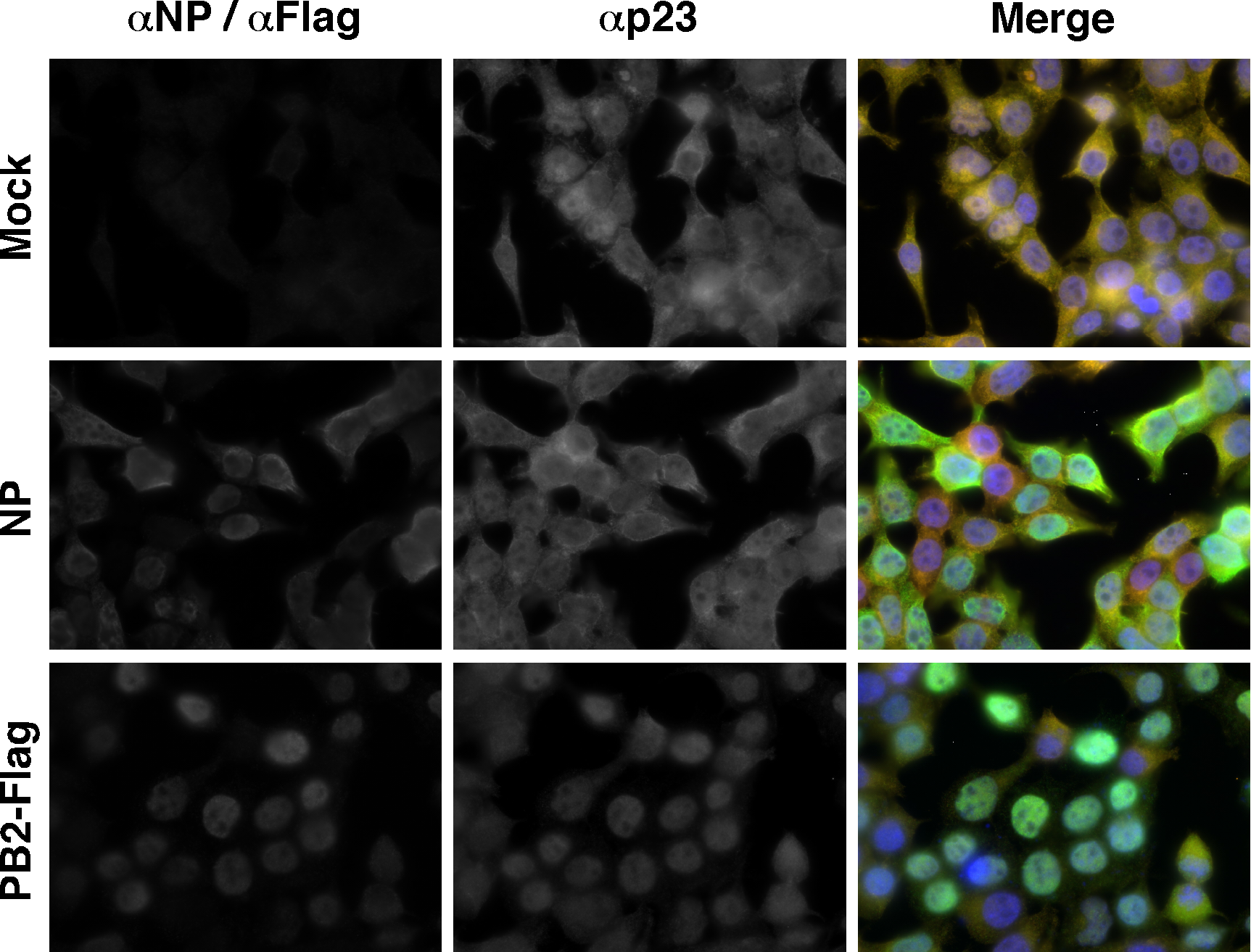

Supplement: Figure S3 — Subcellular localisation of p23 in 293T cells transiently expressing the viral PB2 or NP protein. 293T cells were transfected with a plasmid encoding NP (middle pannel), PB2-Flag (lower panel) or mock-transfected (upper panel). At 24 hpi cells were fixed, permeabilized, and stained with antibodies specific for the Flag tag (PB2) or the NP protein, together with an anti-p23 antibody. Samples were analyzed under a fluorescence microscope (Zeiss Axioplan 2 Imaging - Zeiss ApoTome). A merge of the signals corresponding to DAPI (blue), Flag or NP (green) and p23 (red) is shown. (TIF) [file pone.0023368.s003.tif]
